# Bounded research ethicality: researchers rate themselves and their field as better than others at following good research practice

**DOI:** 10.1038/s41598-024-53450-0

**Published:** 2024-02-06

**Authors:** Amanda M. Lindkvist, Lina Koppel, Gustav Tinghög

**Affiliations:** Department of Management and Engineering, Division of Economics, Linköping University, 581 83 Linköping, Sweden

**Keywords:** Human behaviour, Psychology

## Abstract

Bounded ethicality refers to people’s limited capacity to consistently behave in line with their ethical standards. Here, we present results from a pre-registered, large-scale (N = 11,050) survey of researchers in Sweden, suggesting that researchers too are boundedly ethical. Specifically, researchers on average rated themselves as better than other researchers in their field at following good research practice, and rated researchers in their own field as better than researchers in other fields at following good research practice. These effects were stable across all academic fields, but strongest among researchers in the medical sciences. Taken together, our findings illustrate inflated self-righteous beliefs among researchers and research disciplines when it comes to research ethics, which may contribute to academic polarization and moral blindspots regarding one’s own and one’s colleagues’ use of questionable research practices.

## Introduction

We would like to think that researchers are the pinnacle of objectivity and driven by purely scientific motives. However, researchers are also humans (surprise!) and restricted by the same cognitive boundaries and self-serving motivations as people in general. Over the past decade, there have been widespread discussions about the credibility of scientific claims due to a number of high-profile cases of scientific misconduct, low replication rates across several academic fields^1–4^, and empirical evidence that the use of questionable research practices is surprisingly common^5–7^. To improve scientific research, we need to better understand the social and psychological mechanisms that contribute to the continued use of bad and questionable research practices. The need to explore how external and internal factors influence scientific activities was also recently highlighted in a call for the “psychology of science”^8^. Here, we investigate researchers’ beliefs about the extent to which they, and researchers in their field, follow good research practice, relative to other researchers. Our findings suggest that researchers on average hold inflated beliefs about their own research ethicality and the research ethicality of their field. In other words, researchers are not immune to ordinary psychological processes such as self-enhancement, which influence ethical decision-making.

People do not always behave ethically, even when they intend to do so. For example, we fail to help others in need^9^ and overclaim credit for group work^10^. The concept of *bounded ethicality* has been used to explain these and other phenomena in which there is a gap between people’s intended and their actual ethical behavior^11,12^. Bounded ethicality refers to “the systematic and ordinary psychological processes of enhancing and protecting our ethical self-view, which automatically, dynamically, and cyclically influence the ethicality of decision-making”^12^. Specifically, according to Chugh and Kern’s^12^ model of bounded ethicality, people are motivated to view themselves as ethical, and to uphold this self-view we engage in self-enhancement and self-protection. Which of these two processes are activated at a given time depends on the perceived level of self-threat. If self-threat is low, we engage in self-enhancement, by which we view our ethical behaviors as more ethical than they actually are and our unethical behaviors as less unethical than they actually are. For example, people tend to rate themselves as higher than others on a number of traits associated with being ethical^13,14^, to make overly positive predictions of how ethically they are likely to behave^15^, and to believe that their own moral behavior reflects something about themselves while their immoral behavior is due to circumstances^16^. Self-enhancement increases our positive self-view, but over time may lead to a slippery slope of increasingly unethical behavior as we fail to see the ethical implications of our own decisions.

The tendency to self-enhance and self-protect is also a defining feature of Homo Ignorans (“neglecting man”), which refers to humans’ choice to avoid, neglect, and distort information that poses a threat to one’s identity^17,18^. Enhancing and protecting one’s self-view can have benefits on an individual level, for example, by promoting and protecting one's confidence and self-esteem^19^. However, it can have harmful effects on a collective level, where it may lead to increased polarization between groups, escalating conflicts and undermining cooperation, whether in political, cultural, or academic contexts.

In this study, we investigate whether researchers exhibit a self-enhancing bias in their perceptions of the extent to which they follow good research practice. Specifically, we asked researchers to rate (a) the extent to which they follow good research practice compared to other researchers in their field, and (b) the extent to which researchers in their field follow good research practice compared to researchers in other fields. Given that people have a general tendency to rate themselves as better than others on favorable traits and skills^20,21^ and strive to maintain an ethical self-view^11,12^, we predicted that researchers would rate themselves as following good research practice to a greater extent than other researchers in their field. This hypothesis is in line with results from surveys on research misbehavior and questionable research practices showing higher frequencies for observed behavior than for self-reported behaviors^7^.

We also predicted that researchers would rate researchers within their field as following good research practice to a greater extent than researchers in other fields. This hypothesis is in line with the idea that perceptions of in-group members are closely tied to self-perceptions—extending enhancement tendencies to individuals whom one is invested in^22–24^. In addition, individuals tend to exaggerate the relative importance of reaching the goals of one’s in-group over those of out-groups^25^. These exaggerations of goal importance are associated with the perception that one is justified to cut corners or behave unethically to reach those goals. In-group effects based on gender and seniority have previously been found among researchers, indicating a tendency to apply positive traits to other researchers who share one’s identity to a larger extent than to researchers from out-groups^26^. Here, we focus on shared identities based on academic discipline, which become established over time by learning the discipline-specific set of ways to think about and study the world. These discipline-based social identities strengthen over time by focusing on the similarities within fields and exaggerating the divides between them, resulting in academic silos^27^. Thus, researchers may view researchers in their field as more ethical than other researchers because they are highly identified with their discipline and strive to protect their identity as an (ethical) academic.

## Materials and methods

Our methods, hypotheses, and data analysis plan were preregistered on the Open Science Framework: https://osf.io/f453z.

### Sample and study design

The data were collected in a survey sent out to 33,290 Swedish researchers. The survey was distributed by the public agency Statistics Sweden. We used a total population sampling approach, inviting all individuals who met the following three criteria: 1) are registered in the Swedish population register, 2) have a PhD degree or are currently a PhD student, and 3) are hired at a Swedish university or higher learning facility. This last criterion only included state-funded educational institutions. Invitations to the study were sent out in September 2022 by postal mail and digital mailbox with a link to the web-based survey. Three reminders were sent out. Data collection was stopped in December 2022. Participants were able to view the survey in Swedish or English. In addition to the measures collected for the purposes of this study, the survey contained several different measures relating to ethical research behavior. To ensure readability, the survey underwent an initial pilot phase with a group of researchers who provided feedback. To further refine clarity, the survey underwent a metrological review by Statistics Sweden prior to the commencement of data collection.

In total 11,050 researchers responded, resulting in a response rate of 33.2%. Demographic and occupational variables were accessed from national registries and connected to individual survey responses by Statistics Sweden. Academic field included the 6 OECD categories: Natural sciences, Engineering and Technology, Medical and Health Sciences, Agricultural and Veterinary sciences, Social Sciences, and Humanities and the Arts. As specified in our preregistration, this variable was re-coded into a 4-level factor by including researchers from Engineering and Technology and Agricultural and Veterinary sciences as part of the broader category Natural sciences. Table 1 shows the distribution of the sample between genders, age, academic fields, and employment categories. The sample was close to representative of the sampling frame (i.e., the full population of researchers in Sweden) with regards to these variables, apart from slightly lower response frequencies for younger researchers and PhD students. Supplementary Table S1 shows response rates among different demographic and occupational characteristics.

**Table 1:** Sample characteristics.

Characteristic		N = 11,050
Gender	Male	5822 (52.7%)
	Female	5228 (47.3%)
Age	Mean (SD)	48.4 (12.9)
	Median [Min, Max]	48.0 [24.0, 96.0]
Academic field	Humanities	984 (8.9%)
	Natural sciences	3861 (34.9%)
	Medical and health sciences	2811 (25.4%)
	Social sciences	2584 (23.4%)
	Missing	810 (7.3%)
Employment category	Full professor	2091 (18.9%)
	Associate professor	3153 (28.5%)
	Merit-based employment	619 (5.6%)
	Other	1500 (13.6%)
	PhD student	2665 (24.1%)
	Operational staff	1022 (9.2%)
Data type	Quantitative	6051 (54.8%)
	Qualitative	4010 (36.3%)
	Not empirical	878 (7.9%)
	Missing	111 (1.0%)

Values show n (%) if not otherwise specified. Data type refers to the type of data respondents stated they most frequently handle (i.e., collect, analyze, or report) within their work.

### Measures

After answering a series of questions about research ethics, respondents were presented with a description of good research practice taken from the Swedish Research Council ^28^. The description outlined 8 general rules for good research practice: (1) To tell the truth about one’s research; (2) To consciously review and report the basic premises of one’s studies; (3) To openly account for one’s methods and results; (4) To openly account for one’s commercial interests and other associations; 5) To not make unauthorized use of the research results of others; (6) To keep one’s research organized, for example through documentation and filing; (7) Striving to conduct one’s research without doing harm to people, animals or the environment; and (8) To be fair in one’s judgement of others’ research. After reading this description, respondents were asked two questions: (1) *In your role as a researcher, to what extent do you perceive yourself as following good research practices—compared to other researchers in your field?* (2) *To what extent do you perceive researchers within your field as following good research practices–compared to researchers within other fields?* Each item was rated on a 7-point scale ranging from 1 = *Much less than other researchers* to 7 = *Much more than other researchers*, with 4 = *As much as other researchers* as the midpoint. The full survey is available on the OSF page for the overarching (parent) project (https://osf.io/hw8zf/).

### Data analysis

All analyses were performed using R^29^. Our main analyses consist of two-sided one-sample t-tests (coded as linear regressions) for each of the main measures, with the scale midpoint (i.e., 4) as the reference point. In addition, we ran linear regressions for each of the two main measures, predicting the difference between ratings and the midpoint of the scale by age and a binary coded gender variable. Confidence intervals for regression estimates were bootstrapped using the bootstrap percentile method with 10,000 replications, to address potential issues with non-normality. To illustrate potential differences in the effect between academic fields, we calculated effect sizes and confidence intervals for each field. All analyses specified in the pre-registration were followed without deviations or unreported exclusions. All respondents without missing data for the relevant analyses were included.

### Ethics statement

We consulted the Swedish Ethical Review Authority and it was concluded that research of the kind that is conducted in this project is not covered by the Swedish Ethical Review Act (2003:460) and therefore ethical approval is not required. All methods were carried out in accordance with relevant guidelines and regulations. All participants gave informed consent.

## Results

### Do researchers rate themselves as following good practice more than others in their field?

**Figure 1:**
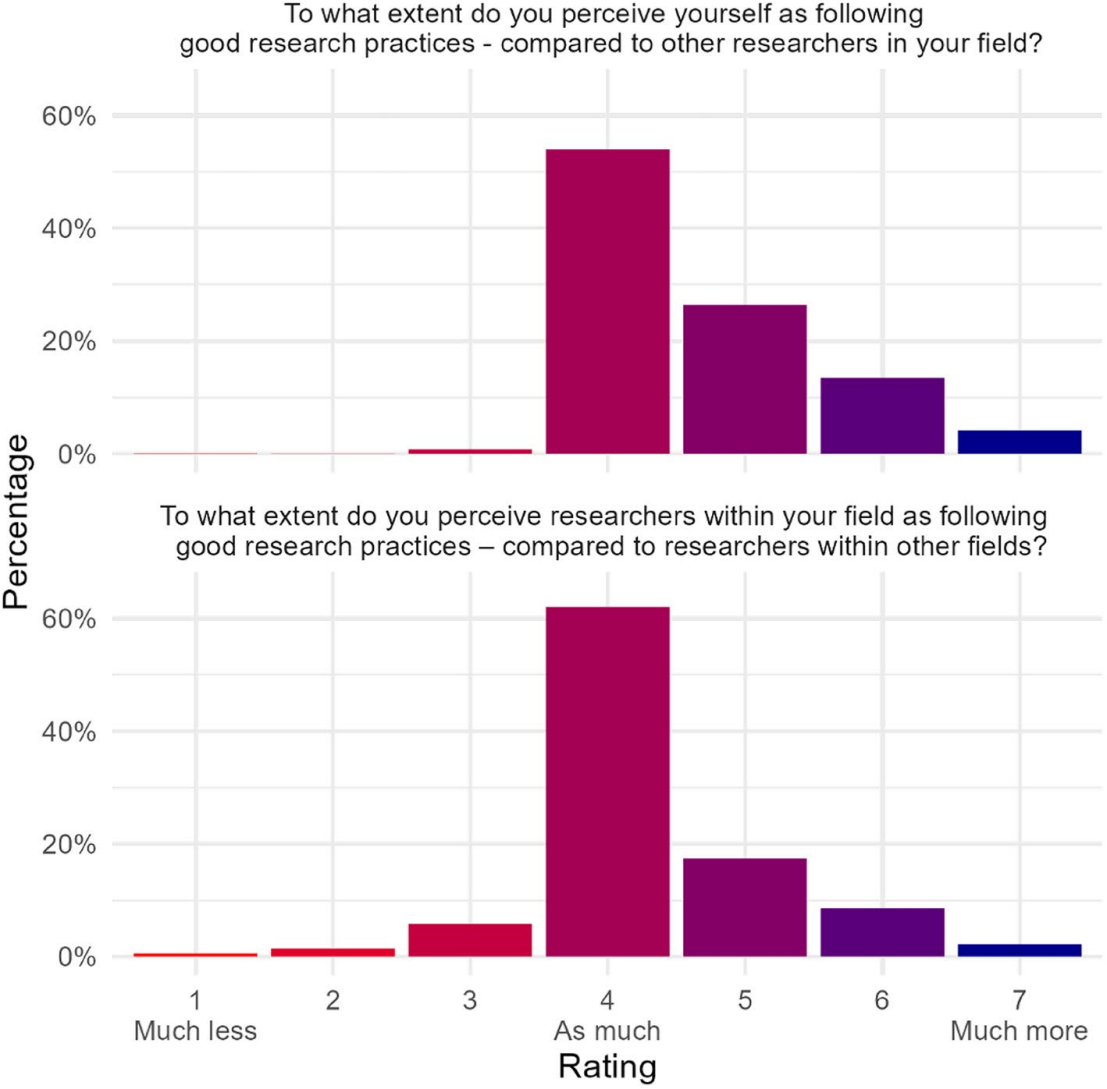
Distributions of ratings of research ethicality. Top panel: comparisons between oneself and researchers in one’s field, *n* = 10,906. Bottom panel: comparisons between researchers in one’s field and those in other fields, *n* = 10,816.

The top panel of Fig. 1 shows the distribution of ratings when comparing oneself to other researchers in one’s field. Although many respondents (55%) rated themselves as following good research practice as much as their peers, practically no one (less than 1% of respondents) rated themselves as following good practice less than their peers. The remainder of the sample (about 44%) rated themselves as following good practice to a greater extent than other researchers in their field. On average, respondents rated themselves as 0.65 scale points higher than the scale midpoint, *t*(10,905) = 77.25, *p* < 0.001. This difference translates into an effect size of Cohen’s *d* = 0.74, 95% CI = [0.70, 0.78]. The effect remained when controlling for age and gender (*B* = 0.65, 95% bootstrapped CI = [0.58, 0.71], *t*(10,903) = 18.78, *p* < 0.001; see Table 2 for full regression output).

**Table 2:** Regression analyses of comparative beliefs about ethical research behavior.

	Self vs. field		Own field vs. other fields	
	(1)	(2)	(3)	(4)
Female		− 0.018		0.056**
		[− 0.051, 0.015]		[0.022, 0.090]
Age		0.000		0.001
		[− 0.001, 0.002]		[− 0.000, 0.002]
(Intercept)	0.652***	0.646***	0.314***	0.239 ***
	[0.635, 0.668]	[0.580, 0.714]	[0.297, 0.331]	[0.167, 0.311]
N	10,906	10,906	10,816	10,816
AIC	28,186.681	28,189.267	28,159.752	28,151.416

This table reports linear regression coefficient estimates, parentheses show 95% bootstrapped confidence intervals (with 10,000 replications). For *self vs. field*, the dependent variable is the distance between ratings of one's own research ethicality and the midpoint of the scale. For *own field vs. other fields*, the dependent variable is the distance between ratings of one's own field research ethicality and the midpoint of the scale. *Female* is a gender dummy. *Age* is the participant’s age in years. AIC: Akaike information criterion. ***p < 0.001. **p < 0.01. *p < 0.05.

**Figure 2:**
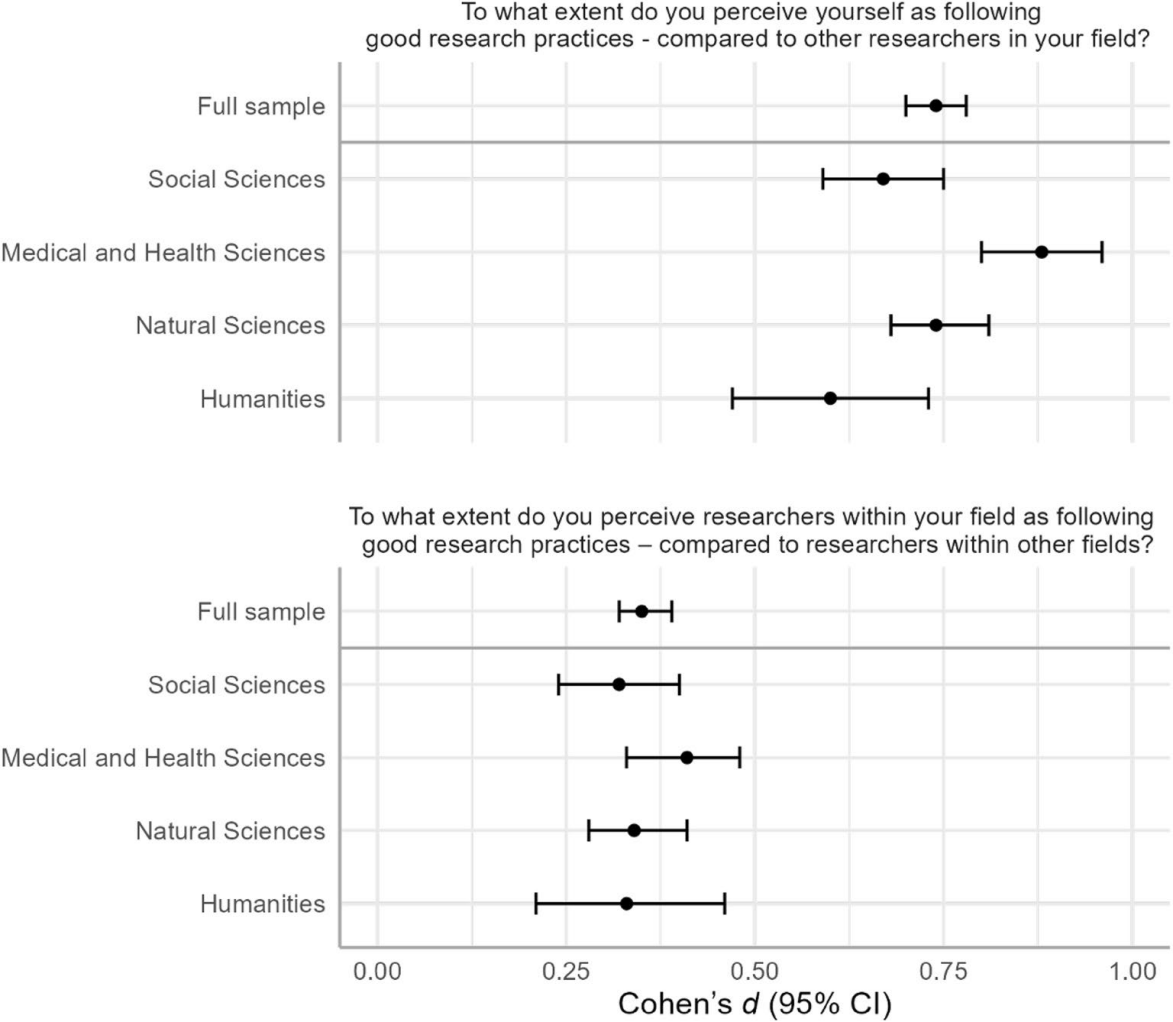
Effect sizes (Cohen’s *d*) with corresponding 95% confidence intervals (CIs) for the full sample and divided by academic field. Top panel: comparisons between oneself and researchers in one’s field. Bottom panel: comparisons between researchers in one's field and in other fields.

The effect was consistent across the four academic fields, varying from a Cohen’s *d* of 0.60 for respondents within Humanities and Arts to a *d* of 0.88 for respondents within Medical and Health sciences (see Fig. 2, top panel). Exploratory analyses of effect sizes at the second level categorization of academic fields showed that all 38 subfields showed a positive effect in the range between Cohen’s *d* 0.50–0.94 (see Supplementary Fig. S2). Note, however, that for 2 of 38 subfields the 95% confidence intervals for *d* crossed 0. Only academic subfields with 30 or more respondents were included into these analyses.

### Do researchers rate researchers in their own field as following good practice more than researchers in other fields?

The bottom panel of Fig. 1 shows the distribution of ratings when comparing researchers in one’s field to researchers in other fields. Again, many respondents (63%) rated researchers in their field as following good practice as much as researchers in other fields, but only a small proportion (about 8% of respondents) rated researchers in their field as following good practice less than researchers in other fields. The remainder of the sample (29%) rated their field as following good practice more than other fields. The overall mean rating was 0.31 scale points higher than the scale midpoint, *t*(10,815) = 36.76, *p* < 0.001, *d* = 0.35, 95% CI = [0.32, 0.39]. The effect remained but was slightly smaller when controlling for age and gender in a linear regression model (*B* = 0.24, 95% bootstrapped CI = [0.17, 0.31], *t*(10,813) = 6.87, *p* < 0.001; see Table 2). The change is explained by females on average giving slightly higher ratings than males (*B* = 0.06, 95% bootstrapped CI = [0.02, 0.09], *t*(10,813) = 3.27, *p* = 0.001). Age was not a statistically significant predictor.

The effect was consistent across the four academic fields, varying between a Cohen’s *d* of 0.32 for respondents in the Social sciences to a *d* of 0.41 for respondents in the Medical and Health sciences (see Fig. 2, bottom panel). Exploratory analyses of effect sizes at the second level categorization of academic fields showed a large degree of variation. While all of the 38 subfields (with 30 or more respondents) showed a positive Cohen’s *d*, the effect size ranged between 0.07 and 0.74 (see Supplementary Fig. S4) and for 16/38 subfields the 95% confidence intervals crossed zero.

As exploratory analyses we also preregistered correlational analysis between ratings of oneself (vs. one's field) and ratings of one's field (vs. other fields), which showed a positive correlation of *r*(10,793) = 0.14, *p* < 001.

## Discussion

We conducted a large-scale survey of 11,050 researchers and found that researchers on average rated themselves as better than other researchers at following good research practice, and rated researchers in their field as better than researchers in other fields at following good research practice. Given that it is statistically impossible for the majority of a group to be better than the group median, our results suggest that researchers on average have inflated beliefs about their own research ethicality and the research ethicality of their field. It is worth noting that many respondents rated themselves as following good research practice *as much as* their peers, thus not self-enhancing relative to others; but the effect we observed occurred on the aggregate level. Importantly, the effect was consistent across all academic fields, although researchers working within Medical and Health Sciences displayed the largest effects both for perceptions about themselves and for perceptions about researchers within their field. Our findings add to the existing literature on the prevalence and predictors of questionable research practices^5–7^, by suggesting that researchers are not immune to ordinary psychological processes such as self-enhancement, which influence the ethicality of decision-making.

According to Chugh and Kern’s^12^ model of bounded ethicality, self-enhancement contributes to the maintenance of an ethical self-view but leads to increasingly unethical behavior over time. Thus, one could speculate that inflated beliefs about one’s research ethicality may lead researchers to underestimate the ethical implications of the decisions they make and to sometimes be blind to their own ethical failures. For example, researchers may downplay their own questionable practices but exaggerate those of other researchers, perhaps especially researchers outside their field. Such distortion of information may be comfortable on an individual level in that it protects one’s (academic) identity, but on a collective level it may contribute to increased academic polarization that hinders constructive discourse, collaboration, and the pursuit of shared knowledge between researchers and academic disciplines. Thus, the finding that inflated beliefs extend to one’s academic discipline could help explain why interdisciplinary collaboration is so difficult to maintain. In addition, self-enhancement may be especially likely to lead to less ethical behavior among researchers who win the “academic game”. That is, researchers who believe they are superior to others in terms of research ethicality may be especially likely to engage in questionable research practices (because they may not see the ethical implications of their behaviors), and these practices are positively reinforced for researchers who also succeed in their career (e.g., who get tenure, publish in prestigious journals, etc.). Furthermore, if we believe ourselves to be more ethical than others in terms of our research practices, then we are less likely to pay attention to information and guidelines aimed at counteracting questionable research practices, because such information and guidelines will appear to be directed to someone else and not to ourselves.

How can people’s inflated ethical self-views be “de-biased” and ethical behavior be increased? According to Chugh and Kern's model of bounded ethicality, people continue to engage in self-enhancement as long as the perceived threat to one’s ethical self-view is low^12^. However, if the perceived self-threat is high, people will engage in self-protective processes, leading them to either behave more ethically (a primary control mechanism) or continue to behave unethically but reframe or justify the behavior (e.g., by placing the responsibility for any negative consequences of the unethical behavior in someone else’s hands; a secondary control mechanism). Thus, one way to increase ethical behavior is to ”nudge” people out of self-enhancement and into self-protection, and, once there, to increase people’s moral awareness (to activate primary rather than secondary control mechanisms). In the context of scientific research, several measures have been proposed (and to some degree implemented) to increase researchers’ moral awareness. These include, for example, affirmative disclosure statements for conflicts of interest^30^ and for methodological practices^31^. Moreover, pre-registration of hypotheses and analysis plans can be one way to constrain researchers' ethical degrees of freedom in a research climate that incentivizes researchers to cut corners to achieve academic success. Overall, structural and cultural changes leading to increased transparency of research practices ought to increase self-threat and, by extension, ethical research behavior.

As with any study, some limitations are warranted. Firstly, although the survey was distributed to all researchers in Sweden via a government agency, we cannot rule out the possibility of self-selection bias. One could speculate that those who chose not to respond might be more likely to rate themselves below average in research ethicality, resulting in an overestimation of the true effect size in the present study. On the other hand, it seems less likely that such self-selection bias would influence ratings of one’s field compared to other fields. Secondly, although the better-than-average effect has been demonstrated in a variety of contexts and is a well-replicated finding^32–35^, it is difficult to extrapolate to what extent responses on the types of scales used in this literature reflect overconfidence or self-serving bias. Benoît and Dubra^36^ argue that a population of completely rational individuals—who accurately update their beliefs in the light of available information—can display beliefs that can be (mis)interpreted as overconfidence or underconfidence. In particular, studies of better-than-average effects rarely ask about the strength of people’s beliefs, which complicates the interpretation of results. Better-than-average effects also tend to be larger for positive (vs. negative) attributes and when using the direct method (by which participants rate themselves compared to an average other on one single response scale) compared to the indirect method (by which participants rate themselves and the average other on two separate scales^34^;). Hence, it is an open question whether we would obtain effects of the same magnitude if we asked about engagement in *un*ethical research practices, relative to others.

The spotlight of the ongoing credibility crisis in science is often on the extreme and clear-cut cases of research misconduct. However, there is a more pressing concern that goes beyond high-profile incidents of research fraud and data fabrication. It pertains to the "everyday" questionable research practices—instances where researchers who want to uphold research ethical principles breach those same principles, often without being aware of it. The current study speaks to this issue. John et al.^6^ refer to questionable research practices as “*the steroids of scientific competition”*. In a world where questionable research practices inadvertently are rewarded, researchers who strictly play by the rules find themselves at a disadvantage. On an everyday basis, researchers are faced with the dilemma of whether they should do what is best for themselves and their career or what is best for scientific progress. Therefore, research ethics should not primarily be about pointing fingers at others, but about looking at oneself in the mirror. We are all boundedly ethical researchers who sometimes breach our own research ethical standards. To restore science’s credibility, we need to create incentive structures, institutions, and communities that foster *ethical humility* and encourage us to be our most ethical selves in an academic system that otherwise incentivizes us to be bad.

### Supplementary Information

Supplementary Information.

## Data Availability

Data for this study are openly accessible at https://osf.io/ku9nd/.

## Code availability

All analysis code needed to reproduce the study’s main analyses are publicly available on the project’s OSF repository (https://osf.io/ku9nd/). In addition, the uploaded HTML-document includes output, packages, and package versions.
