# Supplementary material for: Bounded research ethicality: researchers rate themselves and their field as better than others at following good research practice
Source: Sci Rep. 2024 Feb 6;14:3050. doi: 10.1038/s41598-024-53450-0 (PMC10847100; doi:10.1038/s41598-024-53450-0)
Supplement: Supplementary file 1 — Supplementary Information. [file 41598_2024_53450_MOESM1_ESM.pdf]

Supplementary Information for

Bounded research ethicality: Researchers rate themselves and their field as better than others at following good research practice

Amanda M. Lindkvist, Lina Koppel, Gustav Tinghög

This PDF file includes:

Figures S1 to S4

Table S1

Supplementary Figures

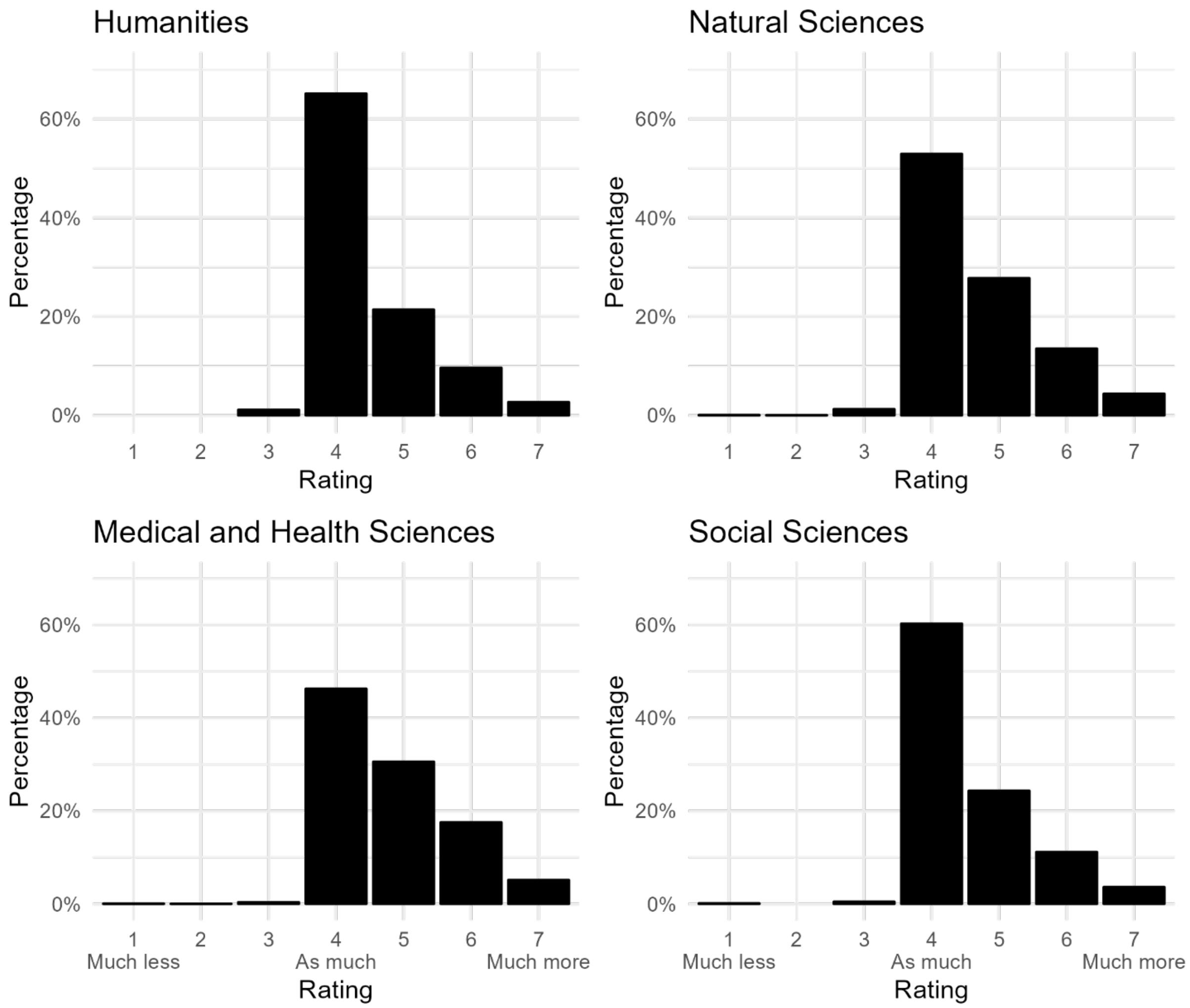

Supplementary Figure S1. Comparisons of ethical research behavior between oneself and researchers in one's field, for each academic field.

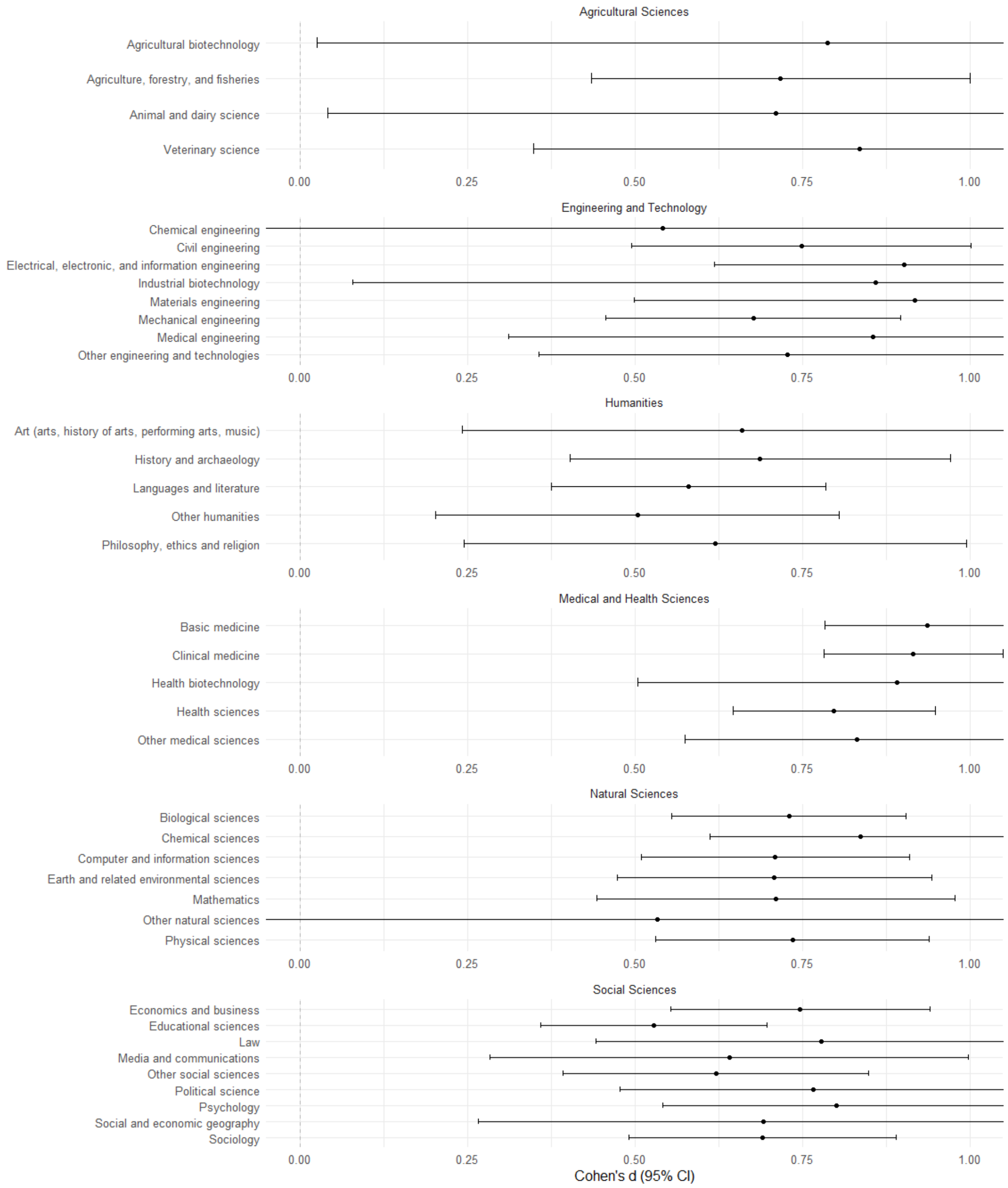

Supplementary Figure S2. Effect sizes (Cohen's *d*) for the difference between self vs. field and the midpoint of the scale with corresponding 95% confidence intervals, for each academic subfield.

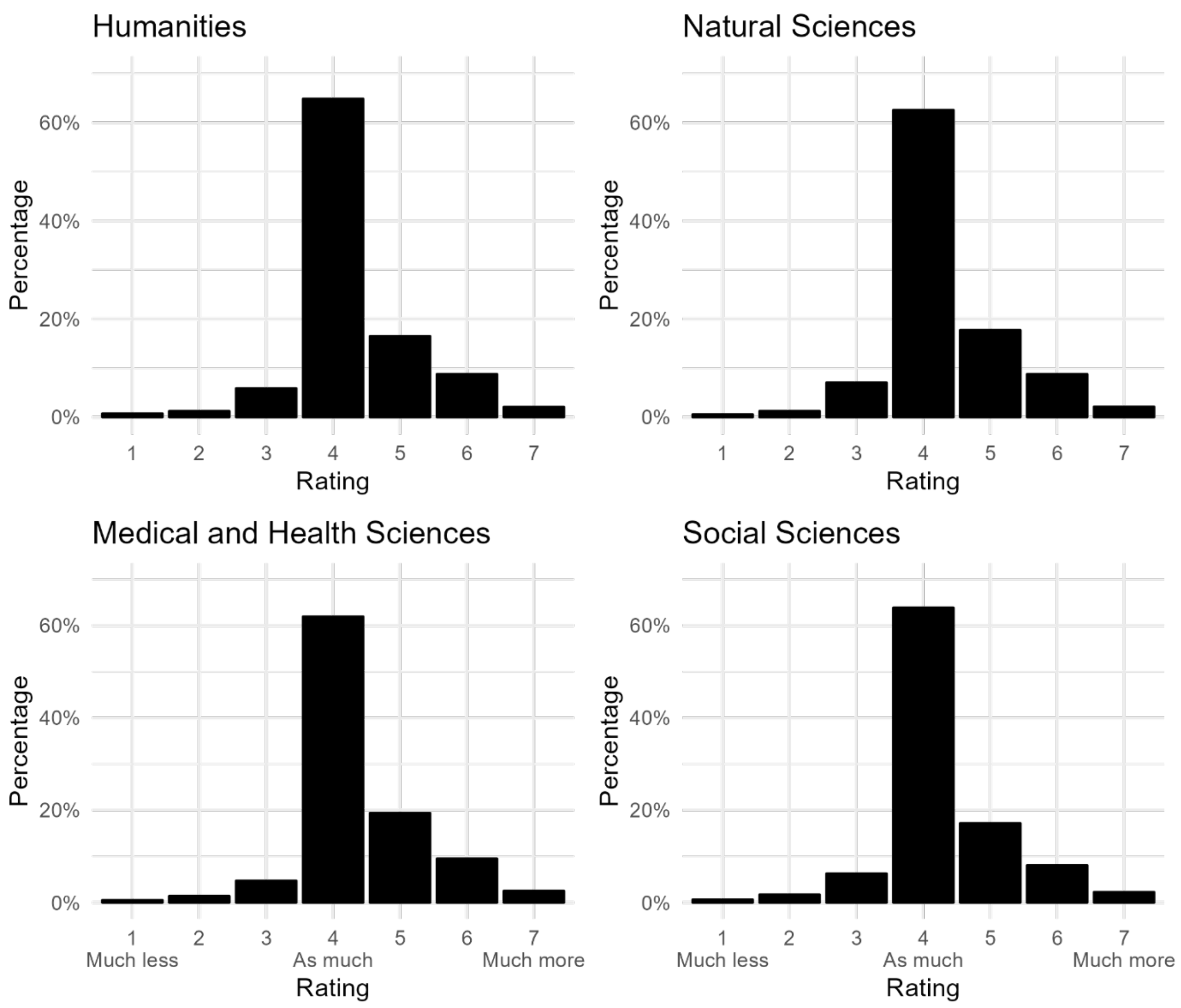

Supplementary Figure S3. Comparisons of ethical research behavior between researchers in one's field and researchers in other fields, for each academic field.

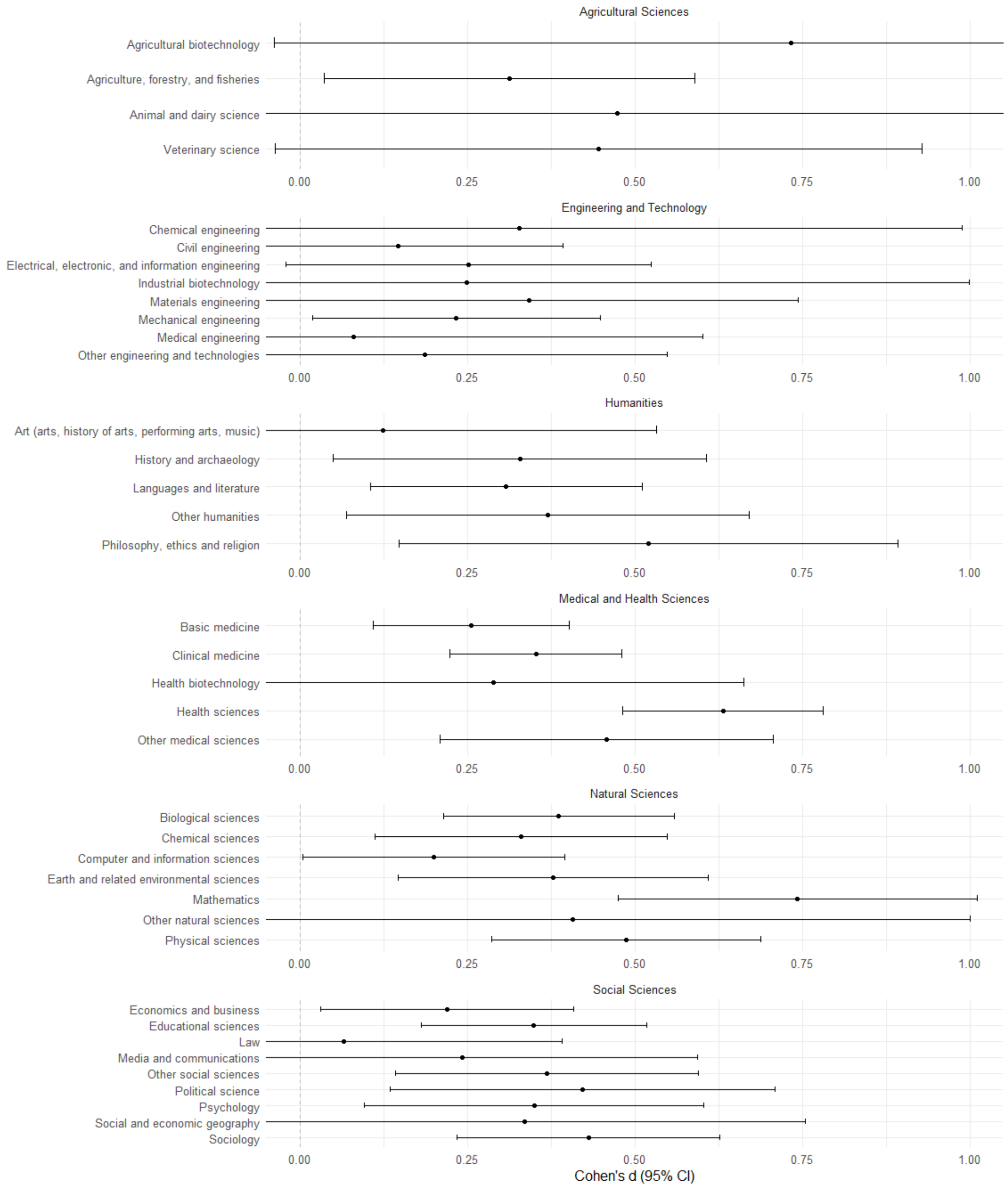

Supplementary Figure S4. Effect sizes (Cohen's *d*) for the difference between own field vs. other fields ratings and the midpoint of the scale with corresponding 95% confidence intervals, for each academic subfield.

Supplementary Tables

Supplementary Table S1. Response rates for different sociodemographic and occupational groups.

	**Response percentage**	**Number of responses**
**Full sample**	33.2%	11050
**Gender**		
Male	33.4%	5822
Female	33.0%	5228
**Age**		
34 years or younger	25.6%	1986
35 – 44	30.8%	2822
45 – 54	34.4%	2711
55 years or older	41.6%	3531
**Education level**		
Post-secondary education shorter than three years *or* Information missing for education level	10.2%	169
Post-secondary education three years or longer	29.2%	2356
Education at the research level (PhD-level education)	36.2%	8525
**Marital status**		
Married	35.2%	6203
Unmarried	31.0%	4847
**Country of birth**		
Nordic Countries	38.0%	8591
Europe excluding the Nordic countries	27.2%	1388
Rest of the world	19.3%	1071
**Municipal groups**		
Metropolitan or metropolitan adjacent municipalities	31.5%	4803
Larger cities or municipalities adjacent to larger city	34.5%	5510
Smaller towns/urban areas and rural municipalities	36.2%	737
**PhD degree**		
Yes	36.2%	8385
No	26.2%	2665
**Academic field**		
Humanities and Arts	32.0%	984
Agricultural Sciences and Veterinary Medicine	38.4%	364
Medicine and Health Sciences	34.8%	2811
Natural Sciences	31.7%	2275
Social Sciences	33.6%	2584
Engineering	30.0%	1222
Information missing for Academic field	36.3%	810

	Response percentage	Number of responses
**University or institution**		
University of Gothenburg	34.2%	1120
Karolinska Institute	32.9%	913
Royal Institute of Technology (KTH)	24.9%	552
Linköping University	37.2%	833
Lund University	33.7%	1515
Stockholm University	33.4%	1011
Umeå University	35.7%	751
Uppsala University	32.3%	1319
Other institutions with less than 1000 researchers	33.4%	3036
**Employment category**		
Lecturers	34.9%	250
Administrative Staff, Library Staff	38.0%	566
Other Research and Teaching Staff	35.4%	1250
Commissioned staff	40.3%	125
Doctoral Students	26.2%	2665
Associate Professors	35.0%	3153
Merit-based employment (incl. Postdocs and Assistant Professors)	32.0%	619
Professors	40.2%	2091
Technical Staff	35.7%	331

Note. The group-wise response percentages should be interpreted in relation to the overall response percentage, reported in the first row of the table. Deviations from the overall response percentage for specific groups indicate higher or lower response rates for that group.
